# Antidiabetic and Antinephritic Activities of Aqueous Extract of* Cordyceps militaris* Fruit Body in Diet-Streptozotocin-Induced Diabetic Sprague Dawley Rats

**DOI:** 10.1155/2016/9685257

**Published:** 2016-05-04

**Authors:** Chungang Liu, Jingjing Song, Meiyu Teng, Xiaoyi Zheng, Xiangmei Li, Yue Tian, Minlian Pan, Yuhuan Li, Robert J. Lee, Di Wang

**Affiliations:** ^1^School of Life Sciences, Jilin University, Changchun 130012, China; ^2^Division of Nephrology, School of Medicine, Stanford University, Palo Alto, CA 94305, USA; ^3^Division of Pharmaceutics, College of Pharmacy, The Ohio State University, Columbus, OH 43210, USA

## Abstract

*Cordyceps militaris* has long been used as a crude drug and folk tonic food in East Asia. The present study aims to evaluate the antidiabetic and antinephritic effects of the aqueous extract of the* Cordyceps militaris* fruit body (CM) in diet-streptozotocin- (STZ-) induced diabetic rats. During four weeks of continuous oral administration of CM at doses of 0.5, 1.0, and 2.0 g/kg and metformin at 100 mg/kg, the fasting blood glucose and bodyweight of each rat were monitored. Hypoglycemic effects of CM on diabetic rats were indicated by decreases in plasma glucose, food and water intake, and urine output. The hypolipidemic activity of CM was confirmed by the normalization of total cholesterol, triglycerides, and low- and high-density lipoprotein cholesterol in diabetic rats. Inhibitory effects on albuminuria, creatinine, urea nitrogen, and n-acetyl-*β*-d-glucosaminidase verified CM's renal protective activity in diabetic rats. Furthermore, CM exerted beneficial modulation of inflammatory factors and oxidative enzymes. Compared with untreated diabetic rats, CM decreased the expression of phosphor-AKT and phosphor-GSK-3*β* in the kidneys. Altogether, via attenuating oxidative stress, CM displayed antidiabetic and antinephritic activities in diet-STZ-induced diabetic rats.

## 1. Introduction

The prevalence of diabetes and metabolic disease is increasing rapidly worldwide and has become a major health problem [1]. Currently, 387 million people are diagnosed with diabetes mellitus, 90% with type 2 diabetes mellitus (T2DM). A deficiency of insulin secretion leads to increased blood glucose levels and organ damage, which further disrupts the metabolism of the three major nutrients, namely, lipids, carbohydrates, and proteins [2, 3]. Various complications including nephropathy, neuropathy, retinopathy, and hyperlipemia are observed in most diabetic patients [4]. The longitudinal data predict that patients with T2DM will have a much more aggressive course of disease with greater risk of early hypertension and nephropathy compared with type 1 (T1DM) patients [5].

Diabetic nephropathy is a major cause of end-stage renal disease with high mortality and morbidity [6]. During the pathogenic process, microalbuminuria follows macroalbuminuria, leading to renal dysfunction. Multiple and complex mechanisms are involved in the pathogenesis of diabetic nephropathy, which is characterized by persistent albuminuria, elevated arterial blood pressure, and a decline in the glomerular filtration rate (GFR) [7]. In 2013, diabetic nephropathy accounted for over 25% of the incidence of end-stage renal disease (ESRD) in the UK, while over 40% of diabetic nephropathic patients in the United States receive dialysis [8].

Current therapy for diabetes focuses only on the recovery of pancreatic islet function and regulation of blood glucose, most of which fails to improve the symptoms of complications [9]. Poorly controlled blood pressure and cholesterol activate inflammatory mediators, and genetic predisposition helps patients progress to an advanced stage of nephropathy. Insulin injection and commonly prescribed drugs such as metformin and pioglitazone produce adverse side effects, including insulin resistance, hypoglycemia, and gastrointestinal disturbances [10]. Due to the limited and unsatisfactory therapeutic effects of antidiabetic agents, alternative medications to treat diabetes and related nephropathy are highly desirable.

Herbs are a source of novel pharmaceuticals not only due to their potent efficacy with fewer side effects, but also due to the complex bioactive compounds they contain [11]. About 1,200 plants have been claimed to have antidiabetic properties, and over 400 plants and their bioactive compounds have been scientifically evaluated for T2DM treatment [12]. Due to its anti-inflammatory, antioxidant, and antitumor activities,* Cordyceps militaris* has been extensively used as a crude drug and folk tonic food in East Asia [13]. In our research group,* Cordyceps militaris* mycelium obtained via submerged fermentation has shown excellent antidiabetic and antinephropathic activities [14]. Aqueous extracts of* Cordyceps militaris* enhance insulin secretion and cholinergic activation in normal Wistar rats [15].

We therefore hypothesized that the polysaccharide-rich aqueous extract of the* Cordyceps militaris* fruit body (CM) may possess antidiabetic and antinephritic properties. A high-fat diet and streptozotocin- (STZ-) induced rat model was used to investigate the effects of CM on diabetes, renal injury, and other underlying mechanisms related to inflammatory factors and oxidative stress.

## 2. Materials and Methods

### 2.1. *Cordyceps militaris* Extract Preparation

**Figure 1:**
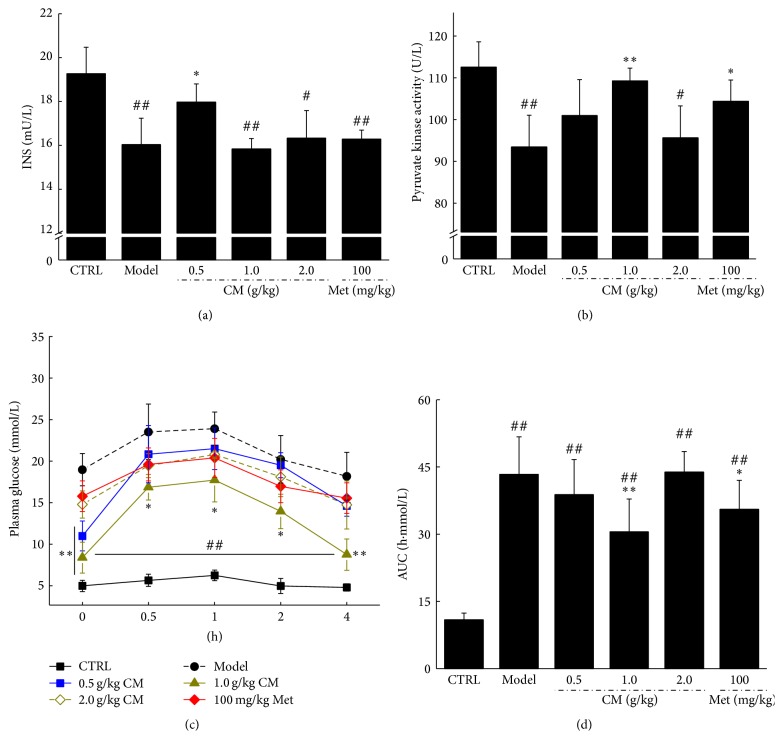
Diet-STZ-induced diabetic rats were treated with or without 100 mg/kg metformin (Met) and* Cordyceps militaris* water extract (CM) for four weeks. After the final drug treatment, the serum levels of insulin (a) and pyruvate kinase (b) were detected in all experimental rats. At the end of the experiment, after an oral treatment of 2 g/kg D-glucose in all experimental rats, the changes of plasma glucose (c) and area under the curve of glucose (d) were analyzed. Data are expressed as mean ± SEM (*n* = 6) and analyzed using one-way ANOVA. ^#^ *P* < 0.05 and ^##^ *P* < 0.01 versus normal controls. ^*∗*^ *P* < 0.05 and ^*∗∗*^ *P* < 0.01 versus nontreated diabetic rats.

*Cordyceps militaris* fruit body (purchased from Qianxiang Co., Ltd., Shenyang, China) was extracted with 10 volumes of double distilled (DD) water at 45°C for 3 h. After centrifugation, the residue was extracted at 80°C for another 3.5 h. After the two extracts were combined, the supernatant was sequentially concentrated in an evaporator under reduced pressure and then freeze-dried to produce a solid aqueous extract (CM). CM contained 29.1% polysaccharides, 20.5% total proteins, 6.1% cordycepic acid, 0.2% adenosine, and 0.4% cordycepin. The concentrations of adenosine and cordycepin were determined using HPLC methods and the results were shown in Figure  1S (in Supplementary Material available online at http://dx.doi.org/10.1155/2016/9685257).

### 2.2. Animal Care

The experimental animal protocol used in the study was approved by the Institutional Animal Ethics Committee of Jilin University. Male Sprague Dawley rats weighing 180–220 g (SCXK(JI)-2014-0003) (purchased from the Norman Bethune College of Medicine, Jilin University, China) were maintained on a 12 h light/dark cycle (lights on 07:00–19:00) at 23 ± 1°C with water and food available* ad libitum*. All efforts were made to minimize animal suffering and reduce the number of animals used.

### 2.3. The Diet-Streptozotocin-Induced Diabetic Rat Model and Drug Administration Procedure

Rats were randomly divided into two groups and fed with either the standard control diet (normal control group, *n* = 6) or a high-fat diet (HFHSD, 12% protein, 5% fat, 67% carbohydrate, 5% cholesterol, and 5% other additives) (*n* = 30) for 8 weeks. HFHSD-treated rats were further intraperitoneally injected with 25 mg/kg STZ agent dissolved in a citrate buffer (0.1 mol/L sodium citrate and 0.1 mol/L citric acid, pH 4.5) for one week (once a day). Rats were defined as diabetic if their blood glucose levels 72 h after the last STZ injection were over 11.1 mmol/L.

Diabetic rats were randomly divided into five groups and orally treated with 2.0 mL/kg sterile saline (HFHSD+STZ diabetic model group, *n* = 6), 0.10 g/kg metformin hydrochloride (Met; from Beijing Jingfeng Zhiyao Co., Ltd, Beijing, China) (Met+HFHSD+STZ group, *n* = 6), and 0.5, 1.0, and 2.0 g/kg CM (CM+HFHSD+STZ group, *n* = 6). Normal rats, which received 2.0 mL/kg sterile saline, served as the normal control group. Over the four-week drug delivery period, bodyweight and blood glucose were recorded weekly. At the end of the experiment, the daily food intake, water intake, and 24 h urine output of each rat were recorded using the diuresis and metabolic cage method.

### 2.4. Oral Glucose Tolerance Test (OGTT) in Rats

After the last drug administration, the rats were fasted for 16 h, before undergoing a glucose tolerance test. Briefly, the rats were weighed and then orally given glucose (2.0 g/kg). Tail-vein blood samples were collected at intervals from 0 to 240 min and assayed via a fast blood glucose meter [16]. The area under the blood glucose curve (AUC) was calculated using the following [17]:

(1) AUC=basal  glycaemia+glycaemia  0.5 h×0.25+glycaemia  0.5 h+glycaemia  1 h×0.25+glycaemia  1 h+glycaemia  2 h×0.5.

### 2.5. Sample Collection and Biochemical Analysis

Before sacrifice, blood was sampled from the heart of each rat under anesthesia. The blood samples were centrifuged at 3000 g for 10 min, and the serum was frozen at −80°C. After sacrifice, the kidneys were collected, and one part was homogenized in DD water (or RIPA buffer) with three washes in ice-cold physiological saline, while the other part was placed in 4% paraformaldehyde for histopathological examination.

The levels were then determined for serum pyruvate kinase (PK), total cholesterol (TC), triglyceride (TG), low-density lipoprotein cholesterol (LDL-C), high-density lipoprotein cholesterol (HDL-C), creatinine (Scr), urea nitrogen (BUN), glutathione peroxidase (GSH-Px), superoxide dismutase (SOD), and n-acetyl-*β*-d-glucosaminidase (NAG) and for albuminuria in urine, malondialdehyde (MDA) in serum and kidneys, and reactive oxygen species (ROS) in kidneys, using commercial kits (Nanjing Biotechnology Co., Ltd., Nanjing, China).

The serum levels of insulin (INS), interleukin-2 (IL-2), interleukin-6 (IL-6), tumor necrosis factor-*α* (TNF-*α*), and 6-keto-PGF were detected using enzyme-linked immunosorbent assay (ELISA) kits (Calbiotech, USA).

### 2.6. Histopathological Examination

The collected kidney tissue was immersed in 4% paraformaldehyde for 48 h and then dehydrated step by step using a gradient of ethanol (50%, 70%, 80%, 90%, 95%, and 100%). Samples were immersed in xylene for 30 min and incubated in paraffin at 65°C overnight. Once embedded in wax, the samples were cut serially into 5 *μ*m thick sections using a microtome (Leica, Germany) and spread over microscopy slides. The sections were deparaffinized with fresh xylene for 10 min, rehydrated with a gradient of ethanol (100%, 90%, 80%, and 70%), and then washed three times with DD water. The sections were analyzed via hematoxylin and eosin (H&E) staining and examined with a light microscope digital camera (Nikon Instruments, Tokyo, Japan).

### 2.7. Western Blot

One part of the kidney tissue was homogenized in a radioimmunoprecipitation assay buffer (RIPA; Sigma-Aldrich, USA) containing 1% protease inhibitor cocktail and 2% phenylmethanesulfonyl fluoride (Sigma-Aldrich, USA). Protein concentrations were determined by the Bradford method, and 40 *μ*g proteins were separated using 10% SDS-PAGE gel and transferred electrophoretically onto nitrocellulose membranes (0.45 *μ*m; Bio Basic, Inc., USA). The transferred membranes were blotted with primary antibodies at 4°C overnight at a dilution of 1 : 1000: phosphor-AKT (ab131443), total-AKT (ab200195), phosphor-GSK-3*β* (ab75745), total-GSK-3*β* (#32391), and glyceraldehyde-3-phosphate dehydrogenase (#2118) (Abcam, Cambridge, UK) and then incubated with horseradish peroxidase-conjugated secondary antibodies (Santa Cruz, USA). Chemiluminescence was detected using ECL detection kits (GE Healthcare, UK). The intensity of the bands was quantified by scanning densitometry using Image J software (National Institutes of Health, Bethesda, USA).

### 2.8. Statistical Analysis

All values were expressed as mean ± SEM. A one-way analysis of variance (ANOVA) was used to detect statistical significance followed by* post hoc* multiple comparisons (Dunn's test) using SPSS 16.0 software (IBM Corporation, Armonk, USA). A value of *P* < 0.05 was considered significant.

## 3. Results

### 3.1. Hypoglycemic Effects on Diabetic Rats

Compared with the normal control group, the diabetic rats clearly consumed more food and water and produced more urine (*P* < 0.01, Table 1). The four-week CM treatment at 1.0 g/kg strikingly decreased their urine output and water intake, and at 0.5 g/kg and 2.0 g/kg food intake was strongly reduced (*P* < 0.01, Table 1).

Reduced bodyweight and elevated blood glucose were observed after STZ treatment (*P* < 0.01, Table 2). Similar to Met, compared with the diabetic model rats, the maximum increase in bodyweight was nearly 31.3% in CM-treated diabetic rats (*P* < 0.01, Table 2). CM at doses of 0.5 and 1.0 g/kg reduced fasting blood glucose by 42.2% and 34.9%, respectively (*P* < 0.05, Table 2). However, only 0.5 g/kg CM clearly increased serum insulin compared with the diabetic model group (*P* < 0.05, Figure 1(a)). Both Met (100 mg/kg) and CM (1.0 g/kg) markedly increased PK activity in diabetic rats (*P* < 0.05, Figure 1(b)).

OGTT was applied to avoid false positive results from fasting blood glucose. Compared with the normal control rats, dramatically higher fasting blood glucose concentrations were noted in the diabetic rats from 0 to 240 min (*P* < 0.01, Figure 1(c)), with 1.0 g/kg CM significantly preventing blood glucose from shooting up at 30 to 240 min (*P* < 0.05, Figure 1(c)). The calculated AUC values for glucose response during the OGTT revealed a striking increment in the diabetic model group (43.3 ± 8.4 h·mmol/L) compared with the normal control group (10.9 ± 1.5 h·mmol/L). CM at 1.0 g/kg and Met at 100 mg/kg showed a significant reduction in AUC (*P* < 0.05, Figure 1(d)).

### 3.2. Hypolipidemic Effects in Diabetic Rats

**Figure 2:**
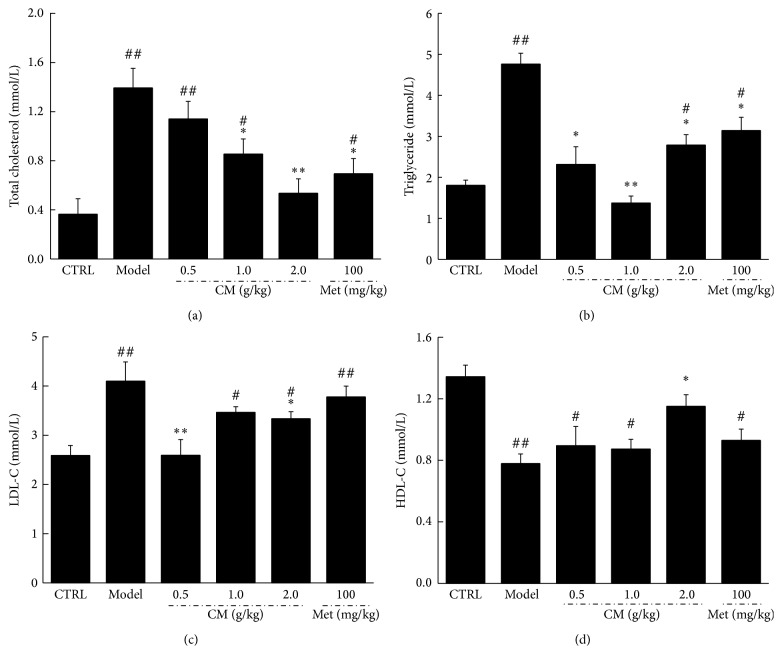
After four-week Met and CM treatment, the serum levels of total cholesterol (a), triglyceride (b), LDL-C (c), and HDL-C (d) in diet-STZ-induced diabetic rats were detected. Data are expressed as mean ± SEM (*n* = 6) and analyzed using one-way ANOVA. ^#^ *P* < 0.05 and ^##^ *P* < 0.01 versus control. ^*∗*^ *P* < 0.05 and ^*∗∗*^ *P* < 0.01 versus model group.

Hyperlipidemia commonly accompanies diabetes mellitus [18]. Thus, a study was carried out to investigate whether CM beneficially affects the abnormal lipid profiles of diabetic rats. As with Met, CM at 1.0 and 2.0 g/kg significantly decreased TC and TG levels (*P* < 0.05, Figures 2(a) and 2(b)). Unlike Met, CM at 0.5 and 2.0 g/kg decreased LDL-C levels in diabetic rats (*P* < 0.05, Figure 2(c)). But only CM at 2.0 g/kg increased HDL-C levels in diabetic rats (*P* < 0.05, Figure 2(d)).

### 3.3. Renal Protection in Diabetic Rats

Albuminuria is traditionally considered a hallmark of diabetic nephropathy [19]. CM strongly suppressed the raised serum albuminuria levels of diabetic rats, especially at 1.0 g/kg (*P* < 0.05, Table 3). Abnormal BUN and Scr levels are recognized manifestations of renal dysfunction, and these were all reduced after four weeks of CM administration (*P* < 0.05, Table 3). However, Met and CM failed to influence serum NGA concentration in diabetic rats (Table 3).

**Figure 3:**
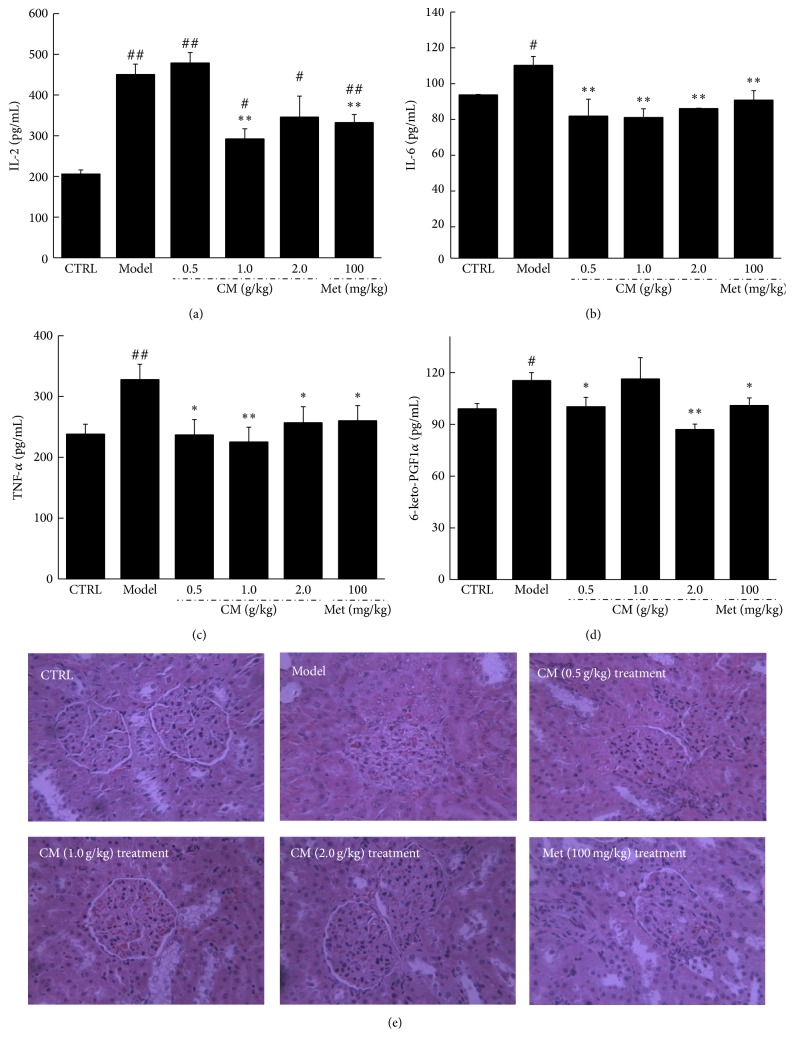
Diet-STZ-induced diabetic rats were orally treated with or without Met and CM at indicated doses for four weeks. The levels of IL-2 (a), IL-6 (b), TNF-*α* (c), and 6-keto-PGF (d) in serum were detected via ELISA method and histopathological changes in kidney collected from all experimental rats were observed through H&E staining (*n* = 6, ×400) (e). Data are expressed as mean ± SEM (*n* = 6) and analyzed using one-way ANOVA. ^#^ *P* < 0.05 and ^##^ *P* < 0.01 versus control. ^*∗*^ *P* < 0.05 and ^*∗∗*^ *P* < 0.01 versus model group.

Hyperglycemia and hyperlipidemia in T2DM always lead to toxicity in the kidneys, inducing renal damage associated with severe inflammation and characterized by the release of multiple inflammatory factors. Extremely high serum levels of IL-2, IL-6, TNF-*α*, and 6-keto-PGF were noted in the diet-induced diabetic rats (*P* < 0.05, Figures 3(a)–3(d)). Met showed a suppressive effect on inflammatory cytokines (*P* < 0.05, Figures 3(a)–3(d)). Compared with the diabetic model group, CM at 1.0 g/kg reduced IL-2 and IL-6 levels by 35.1% and 27.1%, respectively (*P* < 0.01, Figures 3(a) and 3(b)). Additionally, serum TNF-*α* and 6-keto-PGF were reduced in CM-treated diabetic rats by up to 31.2% and 24.6%, respectively (*P* < 0.01, Figures 3(c) and 3(d)). CM treatment also significantly ameliorated the incidence of glomerular basement membrane thickening or mesangial proliferation and of inflammatory infiltrate injuries in the kidneys of diabetic rats (Figure 3(e)).

### 3.4. Antioxidative Effects in Diabetic Rats

Oxidative stress underlies the development of T2DM and related complications [20]. Overproduction of intracellular ROS leads to oxidative stress and deleterious effects on tissues; however, antioxidant enzymes including GSH-Px and SOD prevent oxidative injury. The accumulation of ROS and MDA and low GSH-Px and SOD activity were noted in the serum and/or kidneys of diabetic rats (*P* < 0.05, Table 4). CM enhanced GSH-Px and SOD activity and reduced the serum and kidney levels of ROS and MDA (*P* < 0.05, Table 4). Importantly, CM (2.0 g/kg) decreased ROS production in the kidneys by 12.4% (*P* < 0.05, Table 4). Met was also seen to modulate the oxidative factors in the serum and kidneys of diabetic rats (*P* < 0.05, Table 4).

### 3.5. Activation of AKT/GSK-3*β* in Kidneys

**Figure 4:**
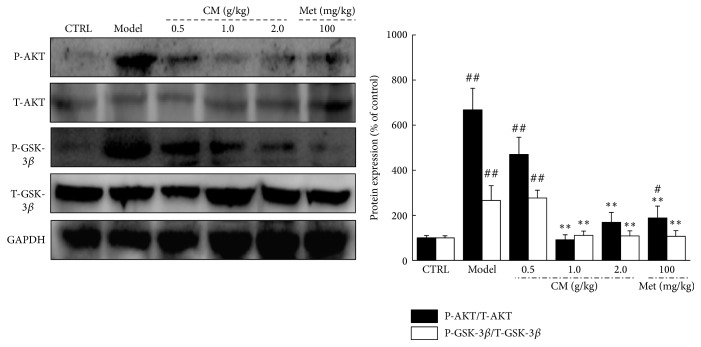
The expressions of T-AKT, P-AKT, T-GSK-3*β*, and P-GSK-3*β* in kidney were analyzed via western blot. Quantification data of the expression of P-AKT and P-GSK-3*β* were normalized by corresponding T-AKT and T-GSK-3*β*, respectively. Data are expressed as mean ± SEM (*n* = 6) and analyzed using one-way ANOVA. ^#^ *P* < 0.05 and ^##^ *P* < 0.01 versus control. ^*∗∗*^ *P* < 0.01 versus model group.

The expression of P-AKT and P-GSK-3*β* in the kidneys of diet-STZ-induced diabetic rats was significantly restored to normal levels after four weeks of CM and Met administration (*P* < 0.01, Figure 4).

## 4. Discussion

The HFHSD-STZ-induced diabetic rat model is closely analogous to the clinical situation of type 2 diabetes mellitus in humans [21]. Combined with the reduction in high fasting blood glucose levels, the modulation of OGTT, a more sensitive measure of early abnormality in glucose regulation [22], further verifies the hypoglycemic activity of CM. Abnormal changes in glucose metabolism are observed in diabetic patients, including decreased glycolysis, impeded glycogenesis, and increased gluconeogenesis [23]. Pyruvate kinase is a key glycolytic enzyme for promoting glucose metabolism and energy production [24]. All of the data support the antidiabetic activity of CM in the diet-STZ-induced diabetic rat model.

Although the pathogenesis of T2DM-induced renal damage is multiple and complicated, dyslipidemia and subsequent lipotoxicity play important roles in this process and accelerate kidney injury. Dyslipidemia, defined as abnormal lipid profiles characterized by increased plasma and tissue levels of TG, TC, and LDL [21], is a major complication associated with high morbidity and mortality in diabetics [25]. Diabetes-related dyslipidemia is responsible for lipid accumulation in the kidney, which leads to insulin resistance, inflammation, and oxidative stress [26]. Gradually, insulin resistance results in the release of adipocytokines and relaxation of the afferent arteriole, finally causing glomerular hyperfiltration, angiogenesis, and mesangial cell proliferation [27, 28]. The antilipemic effect of CM plays an important role in renal protection in HFHSD-STZ-induced diabetic rats.

Oxidative stress has been singled out as a major cause of diabetic complications, especially nephropathy [29]. O^2−^ and nitric oxide (NO) levels are important in kidney and vascular function [30]. ROS, which is responsible for oxidative damage, degrades membrane polyunsaturated fatty acids through sequential peroxidation processes [31] and elevates MDA levels, which serve as biomarkers of tissue oxidative stress [32]. Excessive generation of ROS and MDA in the kidneys leads to tubular obstruction, back-leakage of renal tubules, and contraction of the mesangial cells, finally resulting in the abnormal expression of renal function markers such as Scr, BUN, albuminuria, and NAG [33]. However, cells defend themselves against oxidative stress via the activation of antioxidant enzymes. Antioxidant compounds are a common and effective way to prevent or inhibit pancreatic beta-cell destruction caused by alloxan [34]. SOD catalyzes the conversion of superoxides into hydrogen peroxide and oxygen, while GSH-Px scavenges the hydroxyl radicals [35]. The enhanced activity of SOD and GSH-Px in the serum and kidneys of CM-treated diabetic rats helps to maintain a balance of oxidants and antioxidants by causing the excretion of ROS. Therefore, CM improves renal function by scavenging free radicals, especially ROS and MDA. It is well known that inhibition of AKT phosphorylation downregulates GSK-3*β* phosphorylation [36]. Gardenamide A is reported to attenuate ROS levels by promoting the phosphorylation of AKT, an effect that can be completely abrogated by the AKT inhibitor [37]. In CM-treated diet-STZ-reduced diabetic rats, decreased AKT and GSK-3*β* phosphorylation is responsible for the transcriptional expression of multiple antioxidants to prevent diabetes-related oxidative damage.

Oxidative stress in T2DM favors the appearance of endothelial dysfunction, and oxidative production is an important step in inflammation [38]. Interleukins have important roles during inflammatory development, and the overexpression of IL-2 activates proinflammatory CD4+ T cells, exacerbating the glomerular damage by recruiting macrophages and neutrophils [39]. IL-6, secreted by the glomerular membrane system, is responsible for the proliferation of mesangial cells and the release of inflammatory mediators, including superoxide anions [40]. As reported, TNF-*α* upregulates IL-6 release by the podocytes in coculture with glomerular endothelial cells [41]. Previous studies have reported that oxidative stress is mediated in podocyte apoptosis in the process of diabetic nephropathy [42] and that the progression of renal interstitial fibrosis can be inhibited by suppressing oxidative stress [43]. Thus, CM exerts renal protection in diabetic rats via the regulation of inflammatory factors that are modulated by oxidative stress.

All of the data suggest that CM targets many molecules in the signaling of hyperglycemia, inflammation, and oxidative stress. This “systemic targeting” will completely eliminate the symptoms of diabetes and diabetic nephropathy in a much natural way, so that less adverse effect is expected. As a folk tonic food in China, CM has been emphasizing its safety with few adverse effects. Our subchronic toxic test provides experimental basis for its safety indicating that CM showed no influences on bodyweights (Table 1S), organ indexes (Table 2S), and kidney structures in mice (Figure 2S). On the other hand, the crude drug nature of CM suggests multieffective components, which may show synergistic effect on the disease. It may explain that non-dose-dependent manner was the common way of action of some herbal medicines. Amount of natural productions is reported to show various pharmacological activities via non-dose-dependent manner [44, 45].

There is still a limitation in our present study. Although we confirmed the regulatory effects of Met and CM on inflammatory factors in serum, we failed to detect the related changes in kidney tissues. As reported, Met successfully regulates inflammatory cytokines associated with nephritis but shows no influences on kidney structure [46]. Our further study will focus on the effects of drugs on biochemical indices and pathological changes of kidney.

In summary, we successfully explored the antidiabetic and antinephritic effects of CM in diet-STZ-induced diabetic rats. During the experiment, CM exhibited the ability to reduce blood glucose, decrease blood lipids, reduce renal injury, and lower inflammatory factors through enhanced antioxidant expression and the attenuation of oxidative stress.* Cordyceps militaris* fruit body extract, a safe pharmaceutical agent, thus has great potential as a new treatment for diabetic patients, especially those with nephritis.

## Supplementary Material


*Cordyceps militaris* fruit body was purchased from Qianxiang Co., Ltd., Shenyang, China. Chemical reagents used for biochemical analysis were purchased from Sigma-Aldrich, USA. For histopathological examination, hematoxylin, eosin and other chemical reagents were purchased from Sigma-Aldrich, USA.

## Figures and Tables

**Table 1:** The effects of CM and Met on daily food intake, water intake, and urine output in each experimental rat.

		Food intake (g/100 g)	Water intake (g/100 g)	Urine output (mL/100 g)
CTRL	—	14.0 ± 2.0	14.5 ± 3.6	4.1 ± 1.2
Model	—	19.5 ± 3.2^#^	63.8 ± 6.88^##^	51.1 ± 5.8^##^
CM (g/kg)	0.5	13.3 ± 1.9^*∗*^	42.3 ± 5.9^##*∗*^	40.3 ± 6.7^##*∗*^
	1.0	15.7 ± 1.8	32.6 ± 3.6^#*∗∗*^	30.7 ± 7.1^##*∗∗*^
	2.0	13.6 ± 0.7^*∗*^	37.1 ± 4.2^##*∗*^	39.0 ± 3.6^##*∗*^
Met (mg/kg)	100	16.6 ± 2.1	38.9 ± 6.4^##*∗*^	41.7 ± 6.6^##*∗*^

Daily food intake, water intake, and urine output were normalized to rat body weight, g/100 g or mL/100 g BW. Data are expressed as mean ± SEM (*n* = 6) and analyzed using one-way ANOVA. ^#^ *P* < 0.05 and ^##^ *P* < 0.01 versus normal controls. ^*∗*^ *P* < 0.05 and ^*∗∗*^ *P* < 0.01 versus model group.

**Table 2:** The effects of CM and Met on bodyweight and fasting blood glucose levels in experimental rats.

			Initial	8-week HFHSD feeding	7-day STZ injection	4-week drug treatment			
						1	2	3	4
Body weight (g)	CTRL	—	140.2 ± 25.1	445.3 ± 33.9	468.3 ± 32.8	464.3 ± 29.8	470.3 ± 30.9	480.2 ± 28.4	485.2 ± 32.4
	Model	—	139.2 ± 26.8	450.1 ± 35.9	386.2 ± 35.1^#^	326.0 ± 39.1^##^	315.7 ± 25.9^##^	303.9 ± 38.8^##^	297.0 ± 32.4^##^
	CM (g/kg)	0.5	145.1 ± 29.2	455.3 ± 33.8	381.7 ± 31.5^#^	348.3 ± 32.7^##^	339.2 ± 42.1^#^	347.9 ± 59.2^#^	361.7 ± 27.8^#*∗*^
		1	143.2 ± 30.7	456.3 ± 30.0	388.6 ± 30.5^#^	320.0 ± 42.6^##^	335.7 ± 41.1^#^	355.9 ± 31.9^#^	373.7 ± 30.2^#*∗*^
		2	140.1 ± 25.1	475.1 ± 35.9	385.1 ± 31.9^#^	373.8 ± 23.5^#^	355.2 ± 28.9^#^	345.2 ± 30.7^#^	390.0 ± 22.0^#*∗*^
	Met (mg/kg)	100	139.6 ± 22.1	462.4 ± 32.3	391.9 ± 30.2^#^	326.4 ± 37.9^##^	345.1 ± 32.1^#^	344.0 ± 38.2^#^	349.0 ± 35.6^#*∗*^

Fasting blood glucose (mmol/L)	CTRL	—	4.1 ± 0.7	4.3 ± 0.6	4.6 ± 0.8	4.6 ± 0.7	4.7 ± 0.6	4.7 ± 0.9	4.8 ± 0.7
	Model	—	4.2 ± 0.6	4.6 ± 0.5	18.1 ± 1.5^##^	19.2 ± 1.4^##^	19.1 ± 2.2^##^	21.0 ± 1.9^##^	18.9 ± 1.9^##^
	CM (g/kg)	0.5	4.0 ± 0.5	4.5 ± 0.7	18.3 ± 1.6^##^	15.2 ± 2.1^##^	13.7 ± 1.7^##^	12.3 ± 1.9^#*∗*^	10.8 ± 1.8^#*∗*^
		1	3.9 ± 0.7	4.2 ± 0.9	17.7 ± 1.6^##^	16.1 ± 1.7^##^	13.9 ± 1.4^##^	11.6 ± 1.7^#*∗*^	12.2 ± 1.9^#*∗*^
		2	4.1 ± 0.7	4.5 ± 0.6	18.0 ± 2.1^##^	14.8 ± 1.6^##^	12.5 ± 1.9^##^	11.9 ± 1.9^#*∗*^	12.7 ± 1.7^##*∗*^
	Met (mg/kg)	100	4.2 ± 0.613	4.4 ± 0.7	17.6 ± 2.1^##^	17.1 ± 2.1^##^	14.7 ± 1.6^##^	13.8 ± 1.7^##*∗*^	12.9 ± 1.8^#*∗*^

Data are expressed as mean ± SEM (*n* = 6) and analyzed using one-way ANOVA. ^#^ *P* < 0.05 and ^##^ *P* < 0.01 versus normal controls. ^*∗*^ *P* < 0.05 versus model group.

**Table 3:** The effects of CM and Met on the levels of Scr, BUN, and albuminuria in serum and NAG in urine of diabetic rats.

		Scr (*μ*mol/L)	BUN (mmol/L)	Albuminuria (mg/mL)	NAG (U/L)
CTRL	—	143.8 ± 31.3	5.1 ± 0.5	0.9 ± 0.05	30.2 ± 6.4
Model	—	338.8 ± 32.3^##^	10.2 ± 0.7^##^	2.6 ± 0.3^##^	75.7 ± 7.7^##^
CM (g/kg)	0.5	328.4 ± 22.2^##^	9.2 ± 1.0^##^	2.0 ± 0.3^##^	60.8 ± 7.5^##^
	1.0	228.5 ± 54.6^#*∗∗*^	8.2 ± 0.5^##*∗*^	1.8 ± 0.2^##*∗*^	68.9 ± 8.1^##^
	2.0	226.3 ± 32.1^#*∗∗*^	8.6 ± 1.2^##^	1.9 ± 0.3^##^	73.2 ± 9.0^##^
Met (mg/kg)	100	288.5 ± 74.9^##^	9.3 ± 1.1^##^	2.0 ± 0.3^##^	62.9 ± 9.1^##^

Data are expressed as mean ± SEM (*n* = 6) and analyzed using one-way ANOVA. ^#^ *P* < 0.05 and ^##^ *P* < 0.01 versus normal controls. ^*∗*^ *P* < 0.05 and ^*∗∗*^ *P* < 0.01 versus model group.

**Table 4:** The regulatory effects of CM and Met on the oxidation related factors in serum and kidney of diabetic rats.

		CTRL	Model	CM (g/kg)			Met (mg/kg)
				0.5	1.0	2.0	100
Serum	SOD (U/mL)	245 ± 15	201 ± 12^#^	223 ± 11	248 ± 16^*∗∗*^	237 ± 17^*∗*^	215 ± 17
	MDA (nmol/mL)	8.7 ± 0.8	28.0 ± 0.7^##^	21.9 ± 2.7^##^	12.4 ± 2.7^#*∗∗*^	10.7 ± 1.8^*∗∗*^	15.0 ± 2.5^#*∗∗*^
	GSH-Px (*μ*mol/L)	1116 ± 41	944 ± 37^#^	1000 ± 51	1053 ± 25^*∗*^	994 ± 33^#^	994 ± 9^#^

Kidney	SOD (U/mgprot)	134 ± 14	84 ± 11^##^	110 ± 18^#^	122 ± 13^*∗∗*^	105 ± 14^#^	111 ± 11^#*∗*^
	MDA (nmol/mgprot)	7.0 ± 1.4	11.3 ± 2.6^##^	11.2 ± 2.7^##^	8.2 ± 1.2^*∗*^	9.3 ± 2.0^#^	8.5 ± 1.5^*∗*^
	GSH-Px (*μ*mol/gprot)	6987 ± 318	4925 ± 402^##^	5236 ± 210^#^	6012 ± 462^*∗*^	5985 ± 433^*∗*^	5784 ± 223^#*∗*^
	ROS (FI/gprot)	815 ± 32	988 ± 55^#^	903 ± 31	895 ± 28^*∗*^	865 ± 48^*∗*^	875 ± 36^*∗*^

Data are expressed as mean ± SEM (*n* = 6) and analyzed using one-way ANOVA. ^#^ *P* < 0.05 and ^##^ *P* < 0.01 versus normal controls. ^*∗*^ *P* < 0.05 and ^*∗∗*^ *P* < 0.01 versus model group.
