# Supplementary material for: Antidiabetic and Antinephritic Activities of Aqueous Extract of Cordyceps militaris Fruit Body in Diet-Streptozotocin-Induced Diabetic Sprague Dawley Rats
Source: Oxid Med Cell Longev. 2016 May 4;2016:9685257. doi: 10.1155/2016/9685257 (PMC4870376; doi:10.1155/2016/9685257)

**Supplementary data**

**1. Materials and Methods**

**1.1 *Cordyceps militaris* fruit body extract preparation**

*Cordyceps militaris* fruit body (purchased from Qianxiang Co., Ltd., Shenyang, China) was extracted with 10 volumes of double distilled (D.D.) water at 45°C for 3 h. After centrifugation, the residue was extracted at 80°C for another 3.5 h. The two extracts were combined, and the supernatant was sequentially concentrated in an evaporator under reduced pressure, and then freeze-dried to produce the aqueous extract (CM).

**1.2 Biochemical components analysis**

***1.2.1 Measurement of polysaccharide:*** The amount of total saccharides was determined by anthrone-sulfuric acid method [1]. Monosaccharide concentration was measured by 3,5-dinitrosalicylic acid (DNS) colorimetry [2].

***1.2.2 Measurement of adenosine and cordycepin by high performance liquid chromatography (HPLC)*:** The samples were filtered using a 0.45 µm membrane, and analysed by HPLC with a column Kromasil C18 (4.6 mm × 250 mm, 5 µm particle size) (Waters, USA). The mobile phase was 10 mM KH2PO4, which was dissolved in methanol: distilled water (15:85) and it was driven by a double pump (Waters 150, Millipore, USA). Elution was performed at 30°C with a flow rate of 1 ml/min, and 260 nm UV wavelength was monitored by a tunable absorbance detector (Waters 486, Millipore, USA). All the chemical reagents were obtained from Sigma-Aldrich, USA.

***1.2.3 Measurement of organic acid*:** The amount of organic acid were determined by spectrophotometry method [3].

***1.2.4 Measurement of protein*:** The total protein concentration in samples were analyzed by Kjeldahl methods. [4]

**1.3 Sub-chronic toxicity test**

The experimental protocol was approved by the Animal Ethics Committee of Jilin University. Healthy Kunming mice (Equal numbers of male and female) weighing 18 - 22 g were maintained under a constant 12:12 h light - dark cycle (8:00 am - 8:00 pm) and an environmental temperature of 22 ± 1 ℃. The mice were fed standard chow and given water *ad libitum.* All mice were fed adaptively for one week before experiments.

Mice were randomly assigned into 4 groups (n=20; Equal numbers of male and female), and orally treated with physiological saline (control group; CTRL) and CM at 0.35, 1.05 and 10.5 g/kg for continuous 30 days (Once a day). Bodyweight and food intake were recorded during the whole experiment. Mice were sacrificed by administration of 200 mg/kg pentobarbital at the 30th day, and organs were collected.

**1.4 Histopathological examinations**Kidneys obtained from experimental mice were preserved in 10% neutral-buffered formalin. Histopathological examination was conducted using the routine paraffin embedding technique. Sections of 5 μm thickness stained with hematoxylin and eosin (H&E) were examined under light microscopy for morphological alterations [5].

**1.5 Statistical analysis**

All data were expressed as mean ± S.D.. Statistical significance was determined by one-way analysis of variance (ANOVA) followed by post-hoc multiple comparisons (Dunn’s test) using SPSS 16.0 software (IBM corporation, Armonk, USA) . Values of *P*≤0.05 were considered statistically significant.

**2.** **Results**

**2.1 Concentration of effective constituents in PHC**

CM contained 29.1% polysaccharides, 20.5% total proteins, 6.1% cordycepic acid, 0.2% adenosine and 0.4% cordycepin. The concentration of adenosine and cordycepin were determined using HPLC methods and the results were shown in Fig 1.

**2.2 Effect of CM on bodyweight and organ index in mice**

Subchronic toxicity test was performed to investigate the safety of *Cordyceps militaris* Fruit Body. After 30-day CM administration, compared with control group, all mice displayed normal activities and growth, and no mortality occurred during the experiment. CM-treated mice showed no statistically significant differences on bodyweight compared with control group (Tab. 1). Furthermore, compared with control group, no significant adverse changes on organ indexes including heart, liver, spleen, lung and kidney were observed in CM-treated mice (Tab. 2). Data suggest that CM is safe agent for further experiments.

**2.3 Histologic examination of kidney tissue**

The histopathological changes of kidney after 30-day CM administration were detected via H&E staining. Compared with control group (Fig. 2 A), through observation of glomerulus and the surrounding renal tubules, CM treatment at doses of 0.35, 1.05 and 10.5 g/kg showed no significant changes kidney (Fig. 2 B, C and D).

**References**

[1] A. Leyva, A. Quintana, M. Sanchez, E. N. Rodriguez, J. Cremata, and J. C. Sanchez, “Rapid and sensitive anthrone-sulfuric acid assay in microplate format to quantify carbohydrate in biopharmaceutical products: method development and validation,” *Biologicals,* vol. 36, no. 2, pp. 134-41, 2008.

[2] R. S. Teixeira, A. S. da Silva, V. S. Ferreira-Leitao, and E. P. da Silva Bon, “Amino acids interference on the quantification of reducing sugars by the 3,5-dinitrosalicylic acid assay mislead carbohydrase activity measurements,” *Carbohydr Res,* vol. 363, pp. 33-7, 2012.

[3] C. H. Dong, and Y. J. Yao, “In vitro evaluation of antioxidant activities of aqueous extracts from natural and cultured mycelia of Cordyceps sinensis,” *Lwt-Food Science and Technology,* vol. 41, no. 4, pp. 669-677, 2008.

[4] D. Abrams, D. Metcalf, and M. Hojjatie, “Determination of kjeldahl nitrogen in fertilizers by AOAC official methods 978.02: effect of copper sulfate as a catalyst,” *J AOAC Int,* vol. 97, no. 3, pp. 764-7, 2014.

[5] M. Hutchings, I. Johnson, E. Hayes, A. E. Girling, J. Thain, K. Thomas, R. Benstead, G. Whale, J. Wordon, R. Maddox, and P. Chown, “Toxicity reduction evaluation, toxicity identification evaluation and toxicity tracking in direct toxicity assessment,” *Ecotoxicology,* vol. 13, no. 5, pp. 475-84, 2004.

**Table list**

Table 1 Bodyweights of mice in subchronic toxicity test

| Group | | Body weight (g) | | | | |
| --- | --- | --- | --- | --- | --- | --- |
|  |  | 0d | 7d | 14d | 21d | 30d |
| CTRL | | 19.96±1.15 | 20.92±1.35 | 26.05±1.59 | 29.58±1.96 | 34.53±2.79 |
| CM (g/kg) | 0.35 | 19.50±1.58 | 20.76±1.94 | 26.33±1.70 | 28.98±2.28 | 32.44±2.86 |
|  | 1.05 | 20.14±1.29 | 21.26±1.57 | 26.44±2.44 | 29.81±4.12 | 31.79±3.06 |
|  | 10.5 | 19.85±1.25 | 22.03±2.03 | 25.80±3.15 | 28.73±2.67 | 33.22±2.85 |

Table 2 Organ index of mice in subchronic toxicity test

| Group | | Organ index (g/g) | | | | |
| --- | --- | --- | --- | --- | --- | --- |
|  |  | Heart | Liver | Spleen | Lung | Kidney |
| CTRL | | 0.53±0.05 | 5.75±0.45 | 0.84±0.15 | 0.74±0.10 | 1.52±0.24 |
| CM (g/kg) | 0.35 | 0.53±0.08 | 5.96±0.93 | 0.73±0.14 | 0.75±0.12 | 1.42±0.15 |
|  | 1.05 | 0.56±0.08 | 6.02±0.35 | 0.77±0.32 | 0.72±0.19 | 1.48±0.11 |
|  | 10.5 | 0.53±0.09 | 6.03±0.77 | 0.90±0.49 | 0.72±0.03 | 1.58±0.06 |

**Figure list**

**Fig.1** Adenosine and cordycepin concentration in CM were tested by HPLC method.


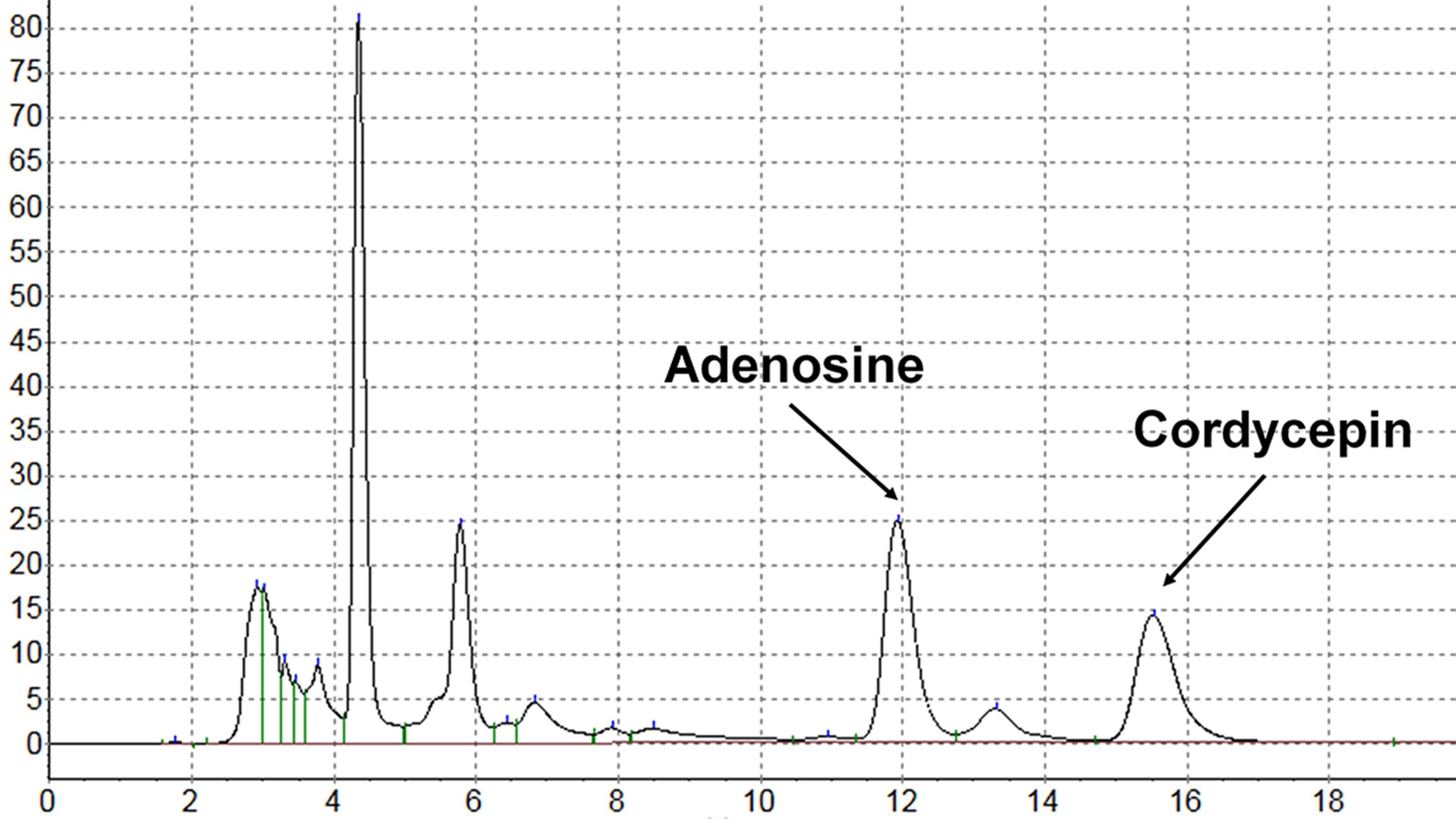


**Fig.2** 30-day CM administration show no histopathological changes on kidney compared with non-treated mice (n=10; x400). (**A**) Control mice; (**B**) 0.35 g/kg of CM-treated mice; (**C**) 1.05 g/kg of CM-treated mice; (**D**) 10.5 g/kg of CM-treated mice.


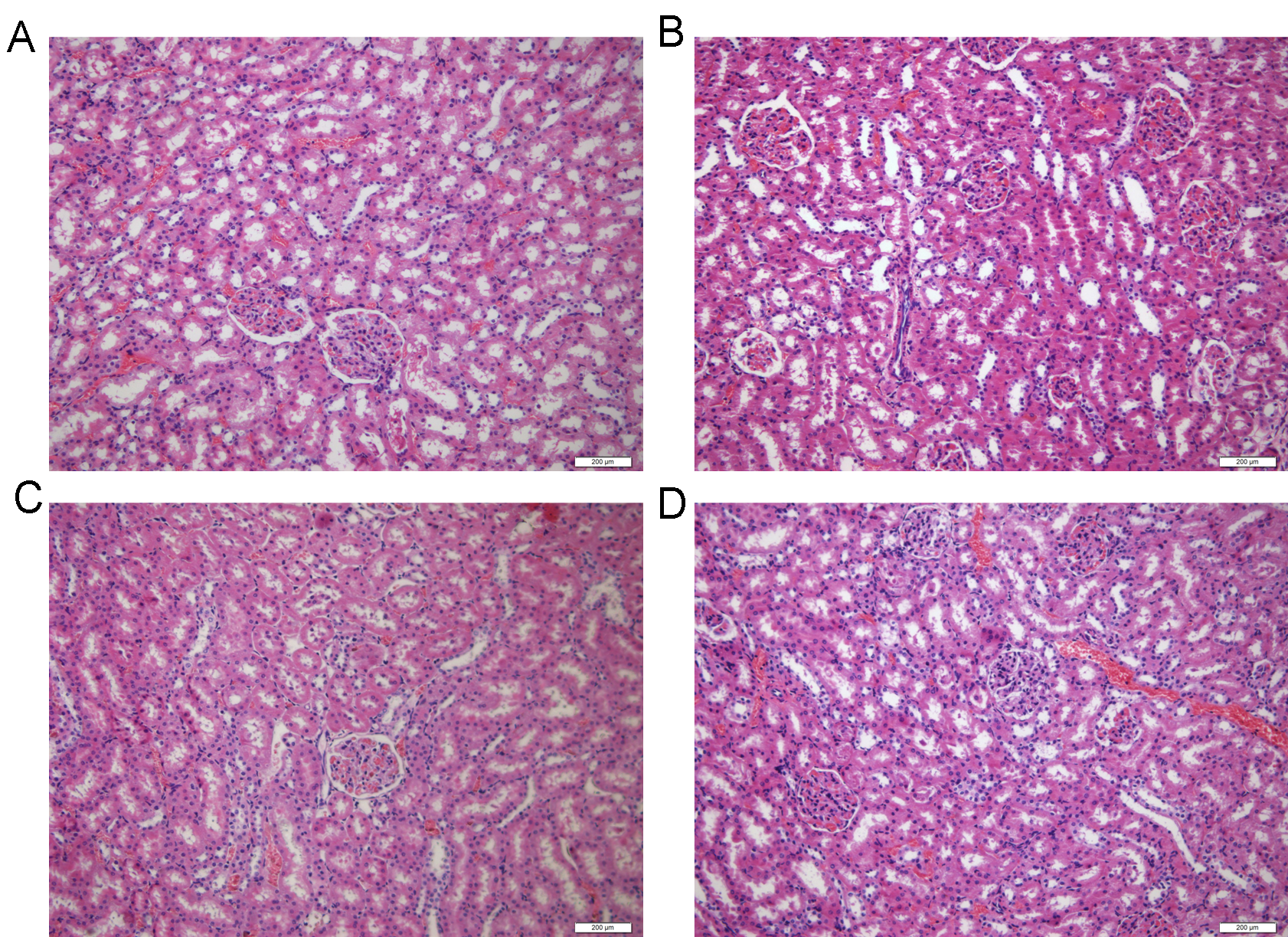

Supplement: Supplementary file 1 — Cordyceps militaris fruit body was purchased from Qianxiang Co., Ltd., Shenyang, China. Chemical reagents used for biochemical analysis were purchased from Sigma-Aldrich, USA. For histopathological examination, hematoxylin, eosin and other chemical reagents were purchased from Sigma-Aldrich, USA. [file 9685257.f1.doc]
